# Trans-umbilical Intragastric Surgery Using Two Alexis Wound Retractors for a Large Trichobezoar in a Four-Year-Old Girl: A Case Report

**DOI:** 10.7759/cureus.43772

**Published:** 2023-08-19

**Authors:** Shojiro Hanaki, Shuichi Katayama, Soichi Nakada

**Affiliations:** 1 Division of Pediatric Surgery, Department of General Surgery, Kurashiki Central Hospital, Kurashiki, JPN

**Keywords:** wound retractor, intragastric surgery, trichophagia, trichotillomania, gastric trichobezoar, gastric bezoar

## Abstract

Trichobezoars are difficult to remove endoscopically and often require surgery. We performed trans-umbilical intragastric surgery using two Alexis wound retractors with successful results in a pediatric patient with a trichobezoar. This method is a safe and cosmetically favorable option for the removal of large trichobezoars and does not require special techniques or instruments. It also contributes to the reduction of postoperative complications such as wound infection and intra-abdominal abscess.

## Introduction

Trichobezoar is a relatively rare disorder, seen in patients with trichotillomania and trichophagia, in which hair ingested orally over a long period of time is solidified by the action of gastric secretions and becomes a mass in the stomach. It is most common in females around puberty and is often recognized by nonspecific symptoms such as abdominal mass, abdominal pain, nausea, and vomiting.

The majority of trichobezoars are removed surgically because large trichobezoars are too large to be removed endoscopically or because it takes too long to crush and remove them [1]. Historically, they have been removed by gastrostomy through a large incision; more recently, laparoscopic surgery using minimally invasive wound closure techniques with wound edge protectors such as the Alexis wound retractor (Applied Medical, Rancho Santa Margarita, California, USA) has been reported [2-4]. However, postoperative complications such as wound infection and intra-abdominal abscesses are still a problem [5].

We report a case in which a large trichobezoar was removed by intragastric surgery using two Alexis wound retractors. This method can be highly recommended for the treatment of pediatric patients because it is safe, simple, and uses the umbilicus as the surgical site, which offers a very favorable cosmetic outcome.

## Case presentation

**Figure 1:**
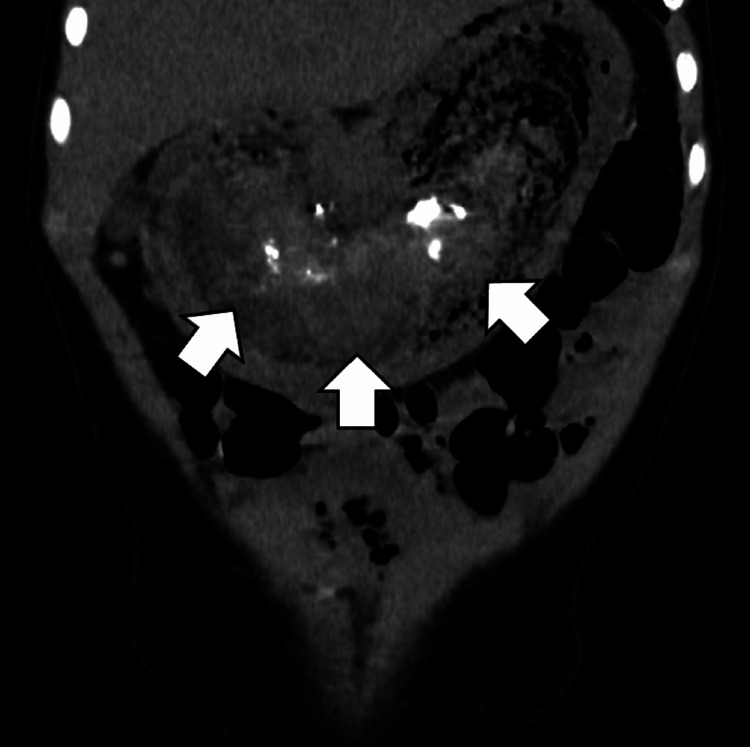
A preoperative coronal abdominal computed tomography scan showed a large bezoar (arrows).

A four-year-old girl presented to our hospital with abdominal pain. Her past medical history was suggestive of trichotillomania and trichophagia. A physical examination revealed a prominent and firm mass in the upper abdomen. An abdominal computed tomography scan revealed a large bezoar (Figure 1).

**Figure 2:**
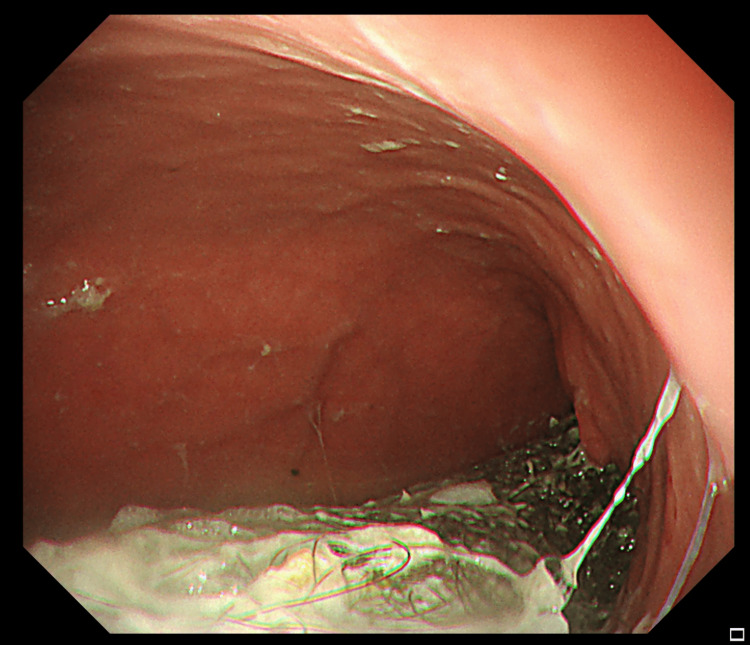
A preoperative upper gastrointestinal endoscopy showed a large trichobezoar.

A diagnosis of trichobezoar was made, and surgical intervention was planned. A preoperative upper gastrointestinal endoscopy was performed under general anesthesia, and the presence of a large trichobezoar was confirmed (Figure 2).

**Figure 3:**
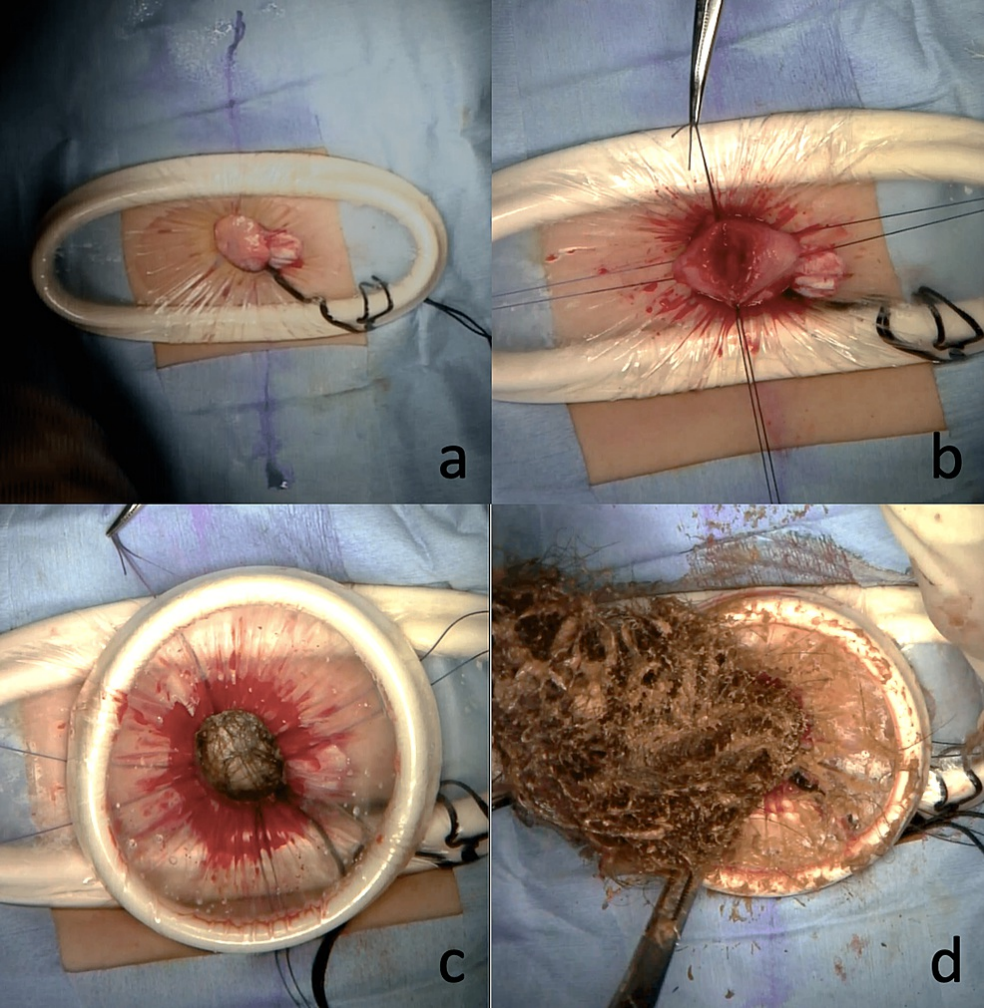
Intraoperative images (a) A 2.0 cm umbilical incision followed by the placement of a small (S)-size Alexis wound retractor (b) A 1.5 cm incision made on the anterior wall of the gastric body (c) An extra extra small (XXS)-size Alexis wound retractor placed in the stomach (d) The trichobezoar was removed by careful shearing with Cooper scissors.

A small 2.0 cm incision was made in the umbilical region, and a small (S)-size Alexis wound retractor was placed (Figure 3a).

A 1.5-cm incision was made in the anterior wall of the gastric body just below the incision site under direct vision to access the lumen. The anterior wall of the gastric body was incised longitudinally, and fixation threads were placed in four directions to prevent tearing of the gastric wall (Figure 3b). An extra extra small (XXS)-size Alexis wound retractor was then inserted into the stomach (Figure 3c). Under direct vision, the trichobezoar was an intricately entangled mass that required careful shearing with Cooper scissors (Figure 3d).

**Figure 4:**
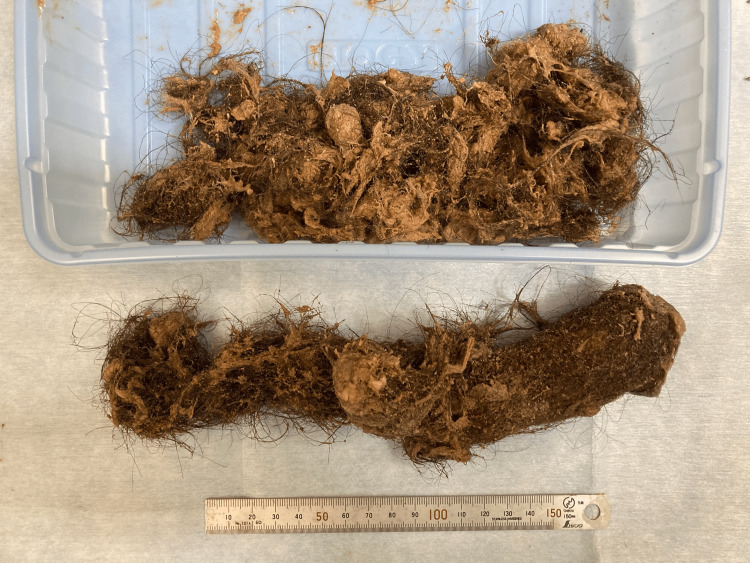
The removed trichobezoar weighing 180 g.

The excised trichobezoar weighed 180 g (Figure 4).

**Figure 5:**
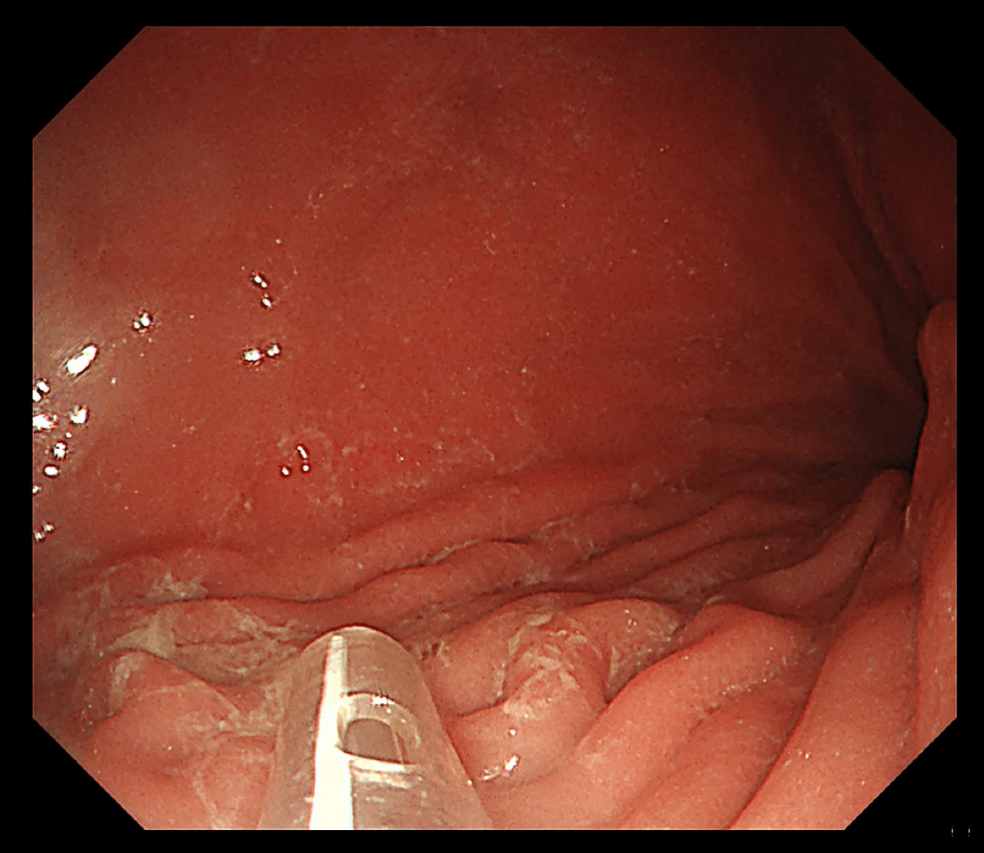
Intraoperative upper gastrointestinal endoscopy showed no residual trichobezoar hairs after trichobezoar removal.

The XXS-size Alexis wound retractor was securely sealed with a surgical glove, and a thorough evaluation of the entire stomach was performed with an upper gastrointestinal endoscope to ensure the absence of any residual trichobezoar hairs (Figure 5).

**Figure 6:**
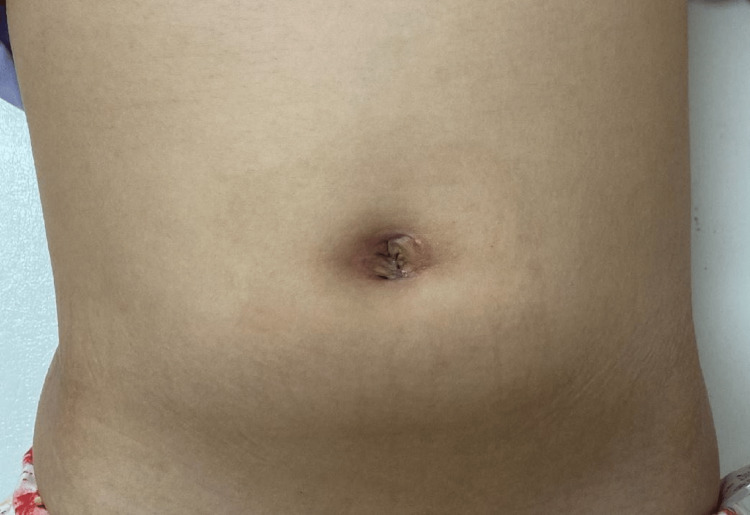
Postoperative appearance of the umbilical site six months after surgery

The gastric incision was closed with Albert-Lembert sutures. After confirming the absence of intra-abdominal contamination, the umbilical incision was closed, marking the completion of the surgical procedure. The operation took 82 minutes with minimal blood loss. The patient was discharged on postoperative day five. At the six-month follow-up, no complications were reported, and there was no recurrence of symptoms (Figure 6).

## Discussion

The primary therapeutic approach for trichobezoars usually involves medical interventions such as lysis and endoscopic treatment [6]. However, due to their large size, most trichobezoars require surgical removal [1]. Coca-Cola lysis has been shown to be effective in dissolving persimmon stones but is generally considered ineffective for trichobezoars, which require endoscopic or surgical removal [1,6]. Although there have been a few reports of successful complete endoscopic removal of small trichobezoars, the majority of cases prove challenging and require surgical intervention [1]. Surgical removal methods including laparotomy, laparoscopy, and intragastric surgery using wound edge protectors such as wound retractors have been reported [2,3]. A more traditional approach, laparotomy, has been widely used in the past but has had problems with surgical wound expansion and contamination [1]. Laparoscopic surgery may improve cosmetic outcomes; however, concerns remain about prolonged operative time and potential intra-abdominal contamination with gastric contents [1,7,8]. Although intragastric surgery using a wound retractor is expected to minimize the surgical wound and reduce the risk of wound infection and intra-abdominal contamination, postoperative wound infection remains a concern [3,4]. In this case, we employed a novel method of using two Alexis wound retractors. To our knowledge, this is the first report of its use in a pediatric patient. In addition, the umbilical incision was made small to minimize the surgical wound.

In this case, a small number of gastric contents was observed to enter between the S- and XXS-size Alexis wound retractors, but the S-size Alexis wound retractor effectively protected the umbilical wound site, preventing contamination and postoperative wound infection. In addition, no intra-abdominal contamination occurred after intragastric surgery. If intra-abdominal contamination is a concern during this procedure, the laparoscope can be used by sealing the S-size Alexis wound retractor with a surgical glove. As demonstrated in this case, the use of two Alexis wound retractors is feasible even for relatively small wounds and can be performed in pediatric cases. There is concern that the small size of the wound may limit gastric delineation, but by sealing the XXS-size Alexis wound retractor with a surgical glove, as in this case, upper gastrointestinal endoscopy can be performed during surgery. With this method, trichobezoars can be completely removed without leaving any residue.

In this case, the preoperative abdominal examination indicated that the stomach could be sufficiently pulled well below the umbilicus, and the technique was successful without complications. If the stomach is located high in the upper abdomen and it is difficult to pull it below the umbilicus, a similar technique can be used by moving the umbilical incision to the upper abdomen using the sliding-window method [9].

## Conclusions

A trans-umbilical intragastric surgery was performed for a large trichobezoar using two Alexis wound retractors. The clinical course was favorable, with no complications. This method is a safe and cosmetically favorable option for the removal of large trichobezoars and does not require special techniques or instruments. It also contributes to the reduction of postoperative complications such as wound infection and intra-abdominal abscess.

## References

[1] Gorter RR, Kneepkens CM, Mattens EC, Aronson DC, Heij HA (2010). Management of trichobezoar: case report and literature review. Pediatr Surg Int.

[2] Tudor EC, Clark MC (2013). Laparoscopic-assisted removal of gastric trichobezoar; a novel technique to reduce operative complications and time. J Pediatr Surg.

[3] Son T, Inaba K, Woo Y, Pak KH, Hyung WJ, Noh SH (2011). New surgical approach for gastric bezoar: "hybrid access surgery" combined intragastric and single port surgery. J Gastric Cancer.

[4] Cundy TP, Brownlee EM, Goh DW, Khurana S (2015). Simplified technique for retrieval of large trichobezoars in children. BMJ Case Rep.

[5] Fallon SC, Slater BJ, Larimer EL, Brandt ML, Lopez ME (2013). The surgical management of Rapunzel syndrome: a case series and literature review. J Pediatr Surg.

[6] Gonuguntla V, Joshi DD (2009). Rapunzel syndrome: a comprehensive review of an unusual case of trichobezoar. Clin Med Res.

[7] Kanetaka K, Azuma T, Ito S, Matsuo S, Yamaguchi S, Shirono K, Kanematsu T (2003). Two-channel method for retrieval of gastric trichobezoar: report of a case. J Pediatr Surg.

[8] Hernández-Peredo-Rezk G, Escárcega-Fujigaki P, Campillo-Ojeda ZV, Sánchez-Martínez ME, Rodríguez-Santibáñez MA, Angel-Aguilar AD, Rodríguez-Gutiérrez C (2009). Trichobezoar can be treated laparoscopically. J Laparoendosc Adv Surg Tech A.

[9] Odaka A, Hashimoto D (2005). Umbilical approach using the sliding-window method to avoid a large abdominal incision: report of two pediatric cases. Pediatr Surg Int.

